# Advances in epigenetics of gastric cancer

**DOI:** 10.3389/or.2026.1656621

**Published:** 2026-01-28

**Authors:** Rihua Zeng, Jianning Chen

**Affiliations:** Department of Pathology, The Third Affiliated Hospital, Sun Yat-sen University, Guangzhou, China

**Keywords:** biomarker, DNA methylation, epigenetics, gastric cancer, histone modification, non-codingRNA

## Abstract

Gastric cancer (GC) persists as a leading cause of global cancer morbidity and mortality, with its pathogenesis intricately linked to epigenetic dysregulation. Emerging research specifies the novelty of these mechanisms—including DNA methylation, histone modifications, non-coding RNAs (ncRNAs), and RNA modifications—in GC initiation, progression, and therapeutic resistance. This review systematically examines key epigenetic mechanisms in GC, dissect the therapeutic implications as diagnostic biomarkers and therapeutic targets. Key insights include (1) aberrant methylation of tumor suppressor genes (e.g., CDH1, RUNX3): in early carcinogenesis; (2) histone lactylation and acetylation modulating immune evasion (3) ncRNAs (e.g., miR-21, HOTAIR); as promising biomarkers; and (4) m6A RNA modification in chemotherapy resistance. We further discuss translational applications of epigenetic biomarkers in liquid biopsies and targeted therapies (e.g., DNMT/HDAC inhibitors). Integrating multi-omics and epigenetic editing technologies may advance precision medicine in GC.

## 1 Introduction

Epigenetic modifications encompass heritable changes in gene activity without altering the DNA sequence, regulating gene expression through mechanisms such as DNA methylation, histone modifications, chromatin remodeling, and non-coding RNA (ncRNA) interactions (1–4). These reversible modifications serve as molecular switches, enabling spatiotemporal control of gene expression and cellular phenotypes. In gastric cancer (GC), epigenetic dysregulation contributes to precancerous lesions, tumor progression, and therapeutic resistance, offering novel avenues for early intervention and personalized therapy.

GC ranks as the fifth most common malignancy globally, with nearly one million new cases annually and a mortality rate ranking fifth among all cancers (5–7). Due to its insidious early symptoms and lack of specific reliable biomarkers, approximately 60% of patients are diagnosed at advanced stages, resulting in a 5-year survival rate below 30%. The role of biomarkers in GC classification has evolved fundamentally, transitioning from irrelevance to central importance. Initially, classification was solely based on morphology (e.g., Lauren classification), with no role for biomarkers. The paradigm shifted with the validation of HER2 as the first actionable predictive biomarker for targeted therapy. The landmark TCGA project in 2014 subsequently established a molecular taxonomy, elevating biomarkers like EBV and MSI to a diagnostic role for defining distinct disease subtypes. In current practice, the role of biomarkers is decisively actionable. The simultaneous testing for a panel of biomarkers (e.g., HER2, PD-L1, MSI) enables clinicians to directly select the most effective targeted or immunotherapy regimens for individual patients. This progression signifies a shift in classification from purely descriptive morphology to a system that directly informs personalized treatment strategies.

Emerging evidence highlights the critical role of epigenetic regulation—including DNA methylation, histone modifications, ncRNAs, and RNA modifications—in GC initiation, progression, and microenvironment remodeling through reversible mechanisms. This review is structured as follows: First, we examine aberrant DNA methylation in early carcinogenesis and progression. Next, we discuss histone modifications, chromatin remodeling, and the roles of non-coding RNAs. We then explore RNA modifications, particularly m6A, in GC pathogenesis. Finally, we evaluate the clinical translation of epigenetic biomarkers and outline future research directions.

## 2 DNA methylation

**FIGURE 1:**
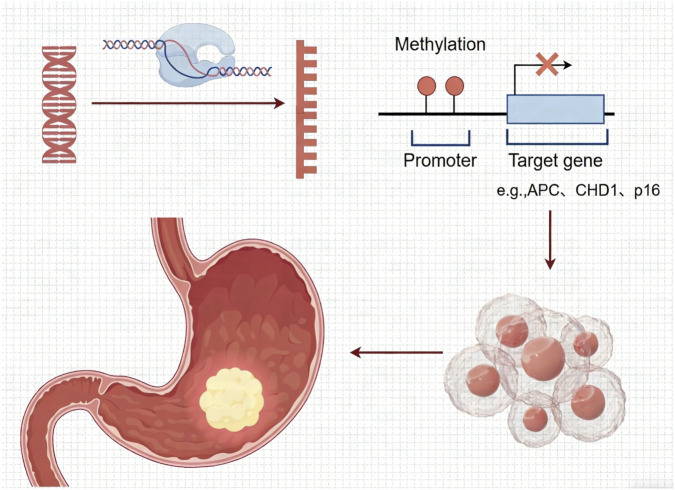
Promoter DNA methylation leads to gene suppression, contributing to the pathogenesis of gastric cancer.

DNA methylation, catalyzed by DNA methyltransferases (DNMTs), involves the addition of a methyl group to cytosine at CpG sites, forming 5-methylcytosine (5-mC), which typically inhibits gene expression in cancer (Figure 1); (8). Aberrant DNA methylation is not only a characteristic of end-stage GC, but also an early and driving event in the pathogenesis of GC.

### 2.1 DNA methylation in early gastric carcinogenesis

In precancerous lesions (such as chronic atrophic gastritis, intestinal metaplasia), promoter hypermethylation of tumor suppressor genes (e.g., p16, MLH1, APC) is observed (9–11). For instance, CDH1 (E-cadherin) methylation occurs in 30%–50% of premalignant lesions, impairing cell adhesion (12). The mechanisms by *Helicobacter pylori* (*H. pylori*) infection activates DNMT1 and inflammatory pathways (e.g., NF-κB), inducing genome-wide methylation aberrations that persist as “epigenetic memory” even after eradication contributes to gastric carcinogenesis are associated with activating DNMT1 and inflammatory signaling pathways (e.g., NF-κB), inducing genome-wide methylation aberrations that persist as “epigenetic memory” even after eradication (13–15). Similarly, EBV infection also shapes the gastric methylome distinctly (16–18). Both pathogens can activate the PI3K/AKT signaling pathway to promote gastric epithelial proliferation (19). Environmental factors (e.g., smoking, diet)can also promote hypermethylation of hub genes including MAPK1 and CDK1 contributing to gastric carcinogenesis (20, 21).

### 2.2 Dynamic DNA methylation changes in tumor progression

Aberrant methylation affects diverse functional gene groups, including tumor suppressors, DNA repair genes, hox genes, and cell cycle regulators collectively driving GC progression (22). Early-stage localized hypermethylation of tumor suppressors like RUNX3 presents opportunities for demethylating agents. In advanced GC, hypomethylation coexists with hypermethylation. For instance, TIMP3 demethylation promotes tumor invasion, whereas PTEN hypermethylation accelerates metastatic progression (23–25). Notably, circulating tumor DNA (ctDNA) methylation in metastatic GC carries metastasis-associated methylation signatures (26).

### 2.3 Summary and future perspectives

Research on DNA methylation in GC has decisively shifted from foundational discovery to clinical translation. Among the plethora of methylated genes reported, a few have emerged with superior merit for specific clinical applications, largely driven by advancements in the last 6 years. For early detection, the most promising and clinically adopted biomarkers are RNF180 and Septin9. Their methylation assays have been endorsed by China’s Expert Consensus and are available as NMPA-approved commercial kits for high-risk population screening (27). For prognostic stratification and monitoring, methylation of SPG20 and FBN1 robustly correlates with poor outcomes, while CPNE1 methylation status shows predictive value for chemotherapy response (28). Technologically, the analysis of EV-derived DNA methylation represents a major innovation for non-invasive detection, while single-cell methylome sequencing is set to resolve tumor heterogeneity (29). In our view, the future lies not in discovering more methylated genes, but in validating and integrating these top-tier candidates (e.g., RNF180/Septin9 for screening, SPG20/FBN1 for prognosis) into multi-analyte liquid biopsy panels to achieve the required sensitivity and specificity for early-stage GC detection (30).

## 3 Histone modifications

**FIGURE 2:**
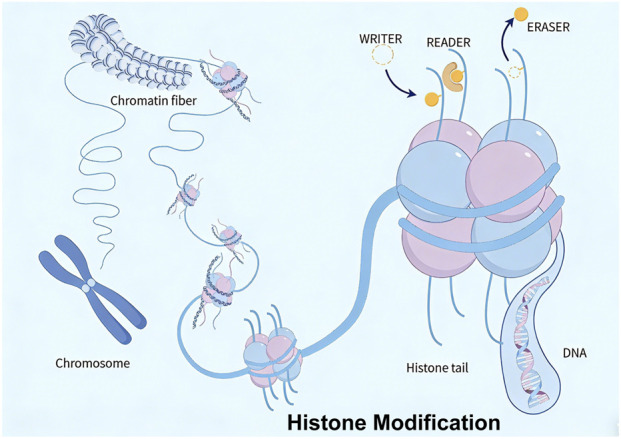
Epigenetic mechanisms of histone modification: acetylation, methylation, lactylation, phosphorylation, and ubiquitination.

Chromatin, composed of DNA, histones, and non-histone proteins, is dynamically regulated by post-translational modifications (e.g., acetylation, methylation, lactylation, phosphorylation, ubiquitination). Histone modifications form a sophisticated regulatory network by exerting crucial effects through coordinated gene expression in GC (Figure 2).

### 3.1 Functional integration of histone modifications

In the GC microenvironment, lactate accumulation (Warburg effect) drives histone H3K18 lactylation (H3K18la), a recently discovered modification that links cellular metabolism directly to epigenetic regulation. The merit of H3K18la as a biomarker stems from its strong correlation with aggressive disease features, including poor prognosis, immune evasion, and metastasis (31–34). Mechanistically, H3K18la facilitates tumor proliferation and metastasis by recruiting M2 macrophages and activating the VCAM1-AKT pathway (35). While the direct detection of histone lactylation in patient blood remains challenging, its upstream driver (lactate) is readily measurable, and the downstream transcriptional signatures it induces (e.g., VCAM1) hold immediate promise as surrogate liquid biopsy biomarkers. This positions lactylation not only as a compelling therapeutic target but also as the conceptual foundation for a new class of metabolic-epigenetic biomarkers.

Acetylation: *H. pylori* carcinogenesis is linked to H4 acetylation in the p21 promoter region (36, 37). H3K9 and H4K16 hypoacetylation strongly associated with poorly differentiated GC (37, 38). Overexpression of histone deacetylases (HDACs) correlates with advanced GC stages may predict immunotherapy response (39, 40).

Methylation: Elevated levels of H3K27, H3K4, and H3K9 methylation collectively promote tumor initiation and progression. The histone methylation regulators EZH2, KDM6A, and KDM6B drive GC susceptibility through synergistic control of H3K27me (41). Lysine-specific demethylase 1 (LSD1) mediated demethylation suppresses antitumor immunity in GC by upregulating exosomal PD-L1 to suppress T-cell responses (42). Histone methyltransferase SETD1A interacts with HIF1α to enhance glycolysis and promote GC progression (43).

Phosphorylation: Histone phosphorylation regulates key processes including DNA repair and apoptosis (44): *H. pylori* infection induces oncogenic H3S10 phosphorylation (45), while overexpression of phosphorylated H3 predicts inferior survival (46).

Ubiquitination: Histone ubiquitination modulates chromatin structure and degradation, with H2B ubiquitination levels correlating with disease progression (47, 48).

Other histone modifications, such as SUMOylation and ADP-ribosylation, also contribute to GC epigenetics, though their roles are less characterized and warrant further investigation.

### 3.2 Summary and future perspectives

Although numerous investigations underscore the significance and therapeutic potential of post-translational histone modifications, their downstream targets remain largely elusive. Consequently, research continues to prioritize the exploration of modification targets and pathways, while clinical translation remains limited.

Technologically, Chromatin Immunoprecipitation with highthroughput sequencing (ChIP-seq) enables genome-wide mapping of epigenetic marks; Cleavage Under Targets and Tagmentation (CUT&Tag) offers a high-resolution technique for profiling histone-DNA interactions; and CUT&Tag with tagmentation-based bisulfite sequencing (CUT&Tag-BS) permits simultaneous detection of histone modifications and DNA methylation, particularly valuable for limited samples. Looking forward, integrating these advanced *in situ* protein-DNA interaction profiling technologies will facilitate systematic elucidation of genome-wide histone modification landscapes and 3D chromatin dynamics in GC.

## 4 Chromatin remodeling

Chromatin remodeling complexes, including SWI/SNF and CHD family members, dynamically regulate nucleosome positioning and chromatin accessibility to drive GC progression. These ATP-dependent complexes function as structural effectors of epigenetic regulation and engage in reciprocal crosstalk with DNA methylation, mutually shaping their genomic localization and activity (49). These structural alterations represent fundamental mechanisms underlying gastric tumorigenesis and present promising targets for epigenetic-based therapeutic interventions.

Key remodeling components demonstrate distinct pathological roles: ARID1A (a SWI/SNF subunit) is frequently mutated in GC, with its loss driving malignant progression, immune microenvironment remodeling, and therapy resistance (50–52). CHD4 enhances chemoresistance by stimulating drug efflux and reducing intracellular cisplatin accumulation, while CHD5 acts as a pan-cancer tumor suppressor (53, 54).

Although SWI/SNF complex mutations have been elucidated more recently than those of classic oncogenes, the remarkable heterogeneity in how SWI/SNF dysfunction drives tumorigenesis presents both a challenge and a unique opportunity for developing precision epigenetic therapies in GC.

## 5 Non-Coding RNAs

**FIGURE 3:**
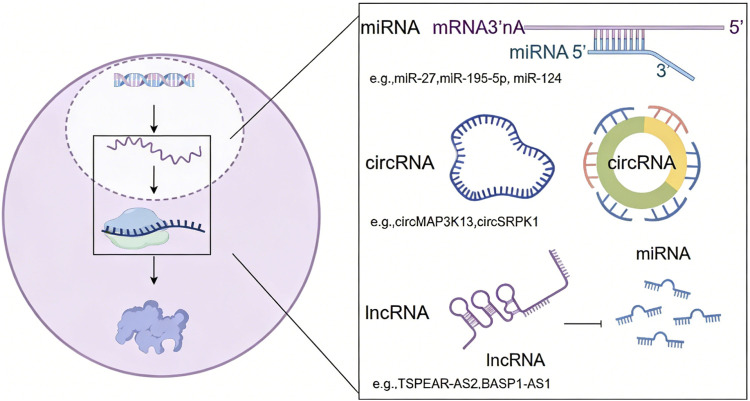
Regulatory functions of non-coding RNAs in GC by modulating gene expression.

Although less than 2% of the human genome encodes proteins, the vast majority of transcripts consist of non-coding RNAs, including microRNAs (miRNAs), long non-coding RNAs (lncRNAs), and circular RNAs (circRNAs). These ncRNAs play crucial roles in GC development by modulating chromatin architecture and gene expression (55, 56); (Figure 3).

### 5.1 miRNAs

In GC, miRNAs regulate gene expression by binding to the 3′untranslated region (3′UTR) of target mRNAs, leading to mRNA degradation or blocking ribosomal translation. Multiple miRNAs (e.g., miR-27, miR-195-5p, miR-124) have been identified as Influencing factors targeting oncogenic pathways such as EMT as well as Wnt/β-catenin signaling pathway in GC (57, 58). These findings highlight the critical regulatory roles of miRNAs in GC pathogenesis and their potential as therapeutic targets.

### 5.2 lncRNAs

The human genome encodes approximately 15,000 lncRNAs characterized by their remarkably diverse mechanisms of action. These regulatory molecules modulate epigenetic modifications through complex interactions suppressing or promoting gastric tumorigenesis (59). For instance, lncRNA TSPEAR-AS2 maintains the stemness of GC stem cells by regulating the miR-15a-5p/CCND1 axis (60). LncRNA BASP1-AS1 mediates histone H3K14 lactylation confering oxaliplatin resistance in GC (61). Despite these advances, significant knowledge gaps remain regarding the precise regulatory mechanisms that lncRNAs employ in GC.

### 5.3 circRNAs

CircRNAs are single-stranded, covalently closed non-coding RNAs produced by back-splicing. They function as miRNA sponges (ceRNAs), modulate protein activity and transcription, and can sometimes be translated into peptides (62). CircRNAs influence GC progression and offer potential as diagnostic markers or therapeutic targets. Such as circMAP3K13 enhances pyroptosis and reduces tumorigenicity and metastasis, circSRPK1 mediates GC progression by interacting with hnRNP A2B1 to regulate RON mRNA alternative splicing (63).

### 5.4 Summary and future perspectives

In current ncRNA research, systematically characterize functionally significant RNA molecules remains a central challenge. Furthermore, ncRNAs form intricate regulatory networks, for which comprehensive understanding remains limited, like lncRNA interact with various histone modification factors to regulate GC development (64, 65). Critical barriers such as low cross-species homology, tumor heterogeneity, and inefficient *in vivo* delivery further impede clinical translation.

To address these challenges, emerging technologies and strategies are advancing the field. Single-cell and spatial transcriptomics enable precise resolution of ncRNA expression dynamics within tissue microenvironments, while artificial intelligence and large language models offer novel computational frameworks for deciphering ncRNA regulatory networks. Clinically, the exceptional stability of ncRNAs in bodily fluids positions them as promising biomarkers.

## 6 RNA modifications

**FIGURE 4:**
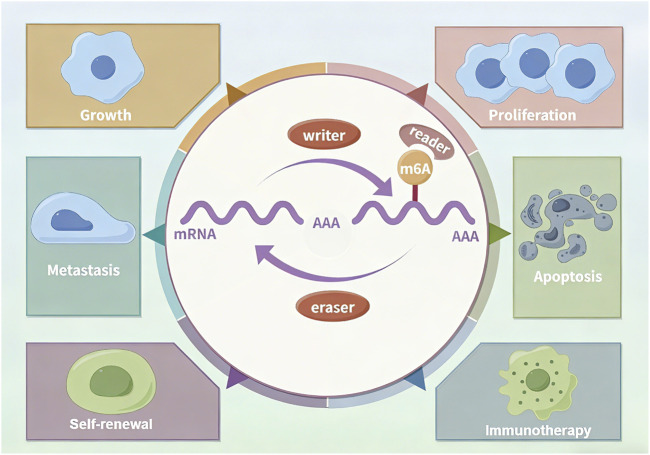
The m^6^A Modification: a representative example of RNA modifications.

RNA modifications play pivotal roles in GC progression by dynamically regulating RNA stability, splicing, and translational efficiency. RNA modification is coordinately regulated by methyltransferases (“Writers”), demethylases (“Erasers”), and binding proteins (“Readers”), collectively governing diverse malignant behaviors in GC (66–68). Research on m6A modification has dominated the field, whereas progress in understanding many other RNA modifications has lagged behind (Figure 4).

### 6.1 The m6A RNA methylation

The m6A writer METTL3 acts as an oncogene by stabilizing STAT5A and DEK mRNAs, thereby promoting GC progression (69–71), while METTL14-mediated m6A modification suppresses GC progression by regulating miR-30c-2-3p/AKT1S1 axis or lnc-PLCB1/DDX21 axis (72, 73). And METTL16 promotes GC via m6A-modification on FDX1 mRNA (74). Meanwhile reader diversify m6A-mediated phenotypes: YTHDF1 enhances USP14 translation to accelerate tumor growth, and YTHDF2 maintains cancer stemness by regulating ONECUT2 degradation, leading to oxaliplatin resistance (75, 76). Reader hnRNPA2B1 contribute to the maintenance of stemness property via Wnt/β-catenin pathway and exacerbate chemoresistance in GC (77). IGF2BP2-meidated m6A of CSF2 in reprogramming mesenchymal stem cells (MSCs), which presents a promising therapeutic target for GC (78). Eraser ALKBH5 suppresses GC by suppressing PI3K/Akt/mTOR signaling through PKMYT1/WRAP53 m6A demethylation yet promotes GC by activating JAK1 signaling via JAK1 demethylation (79–81). Additionally FTO facilitates metastasis by demethylating m6A from caveolin-1 and circFAM192A (82, 83). This regulatory complexity reveals the therapeutic potential of targeting specific m6A regulators in GC.

### 6.2 Other RNA methylations

The m7G modification, driven by the METTL1/WDR4 complex, promotes GC progression through enhancing oncogenic tRNA expression and facilitating the translation of oxidative phosphorylation-related genes, thereby accelerating metabolic reprogramming (84). Clinically, prognostic signatures based on m7G-associated lncRNAs show significant value in predicting patient survival and immunotherapy response (85, 86). Similarly, m5C modifications mediated by writers such as NSUN2 contribute to gastric carcinogenesis, with PTEN mRNA m5C modification playing a key role in progression (87). Elevated m5C levels enhance tumor cell invasiveness, highlighting its potential as a therapeutic target (88, 89).

### 6.3 Other RNA modifications

Upregulation of NAT10 by H. pylori infection promotes gastric tumorigenesis through ac4C-mediated stabilization of the oncogenic transcripts MDM2 and HK2 (90, 91). Additionally, NAT10 enhances the hypoxia tolerance of GC cells by mediating ac4C modification on SEPT9 mRNA, which activates the HIF-1 pathway and glycolytic reprogramming. Combining Apatinib with ac4C inhibition presents a promising strategy to overcome resistance to anti-angiogenic therapy (92, 93).

### 6.4 Summary and future perspectives

The “Writer-Eraser-Reader” network of RNA modifications presents a rich source of biomarkers and therapeutic targets. When evaluating their merit as biomarkers, the key regulators themselves often show the most immediate promise due to their druggable nature and measurable expression changes. For instance, the overexpression of the writer METTL3 and the reader YTHDF1 consistently correlates with poor survival and advanced disease stage, making them strong prognostic biomarkers (69, 75). The eraser FTO is another key player, whose upregulation promotes metastasis and could serve as both a prognostic marker and a therapeutic target (82, 83). The past 6 years have been pivotal in moving from cataloging these modifications to understanding their functional circuits. The current challenge is no longer just discovery but the development of high-throughput methods for absolute quantification of modifications like m6A in clinical samples. We anticipate that in the near future, panels combining the expression levels of key regulators (e.g., METTL3, YTHDF1, FTO) with specific modification signatures on oncogenic transcripts will provide powerful tools for GC diagnosis and subtyping.

## 7 Clinical translation and future perspectives

### 7.1 Integrated epigenetic networks and biomarker potential

Emerging evidence reveals that DNA methylation and histone modifications frequently co-occur, forming interconnected epigenetic networks that mutually reinforce regulatory outcomes through cell context-dependent molecular interactions (94). This crosstalk is exemplified in GC cell line AGS, where H3K27me3-marked promoter regions consistently exhibit DNA hypermethylation, suggesting the potential for combined epigenetic targeting strategies (95). The development of diagnostic models incorporating both DNA methylation and histone modification profiles in premalignant lesions holds particular promise (96).

CircRNAs have emerged as attractive biomarker candidates due to their unique covalently closed circular structure, which confers exceptional resistance to exonuclease and RNase R degradation while enabling stage-specific expression patterns across diverse tissues (97). Their remarkable stability in biological fluids makes them ideal for liquid biopsy applications.

Epigenetic-based strategies for GC management have demonstrated remarkable clinical potential across multiple applications. For early detection, China’s Expert Consensus on Early Gastric Cancer Screening Technologies has endorsed RNF180/Septin9 methylation assays as viable screening tools, with NMPA-approved commercial kits now available for high-risk populations (27). Additional promising non-invasive biomarkers including MINT31 and APC methylation are under active investigation (98, 99). Prognostically, hypermethylation of SPG20 and FBN1 has shown significant correlation with poor clinical outcomes, while CPNE1 methylation status serves as a predictive biomarker for chemotherapy response (28).

### 7.2 Epigenetic-targeted therapies in GC

Several epigenetic inhibitors have shown promise in preclinical and clinical studies for GC. DNMT inhibitors (e.g., azacitidine) and HDAC inhibitors (e.g., vorinostat, chidamide) are being evaluated for their ability to reverse aberrant methylation and acetylation patterns (100–102). Additionally, inhibitors targeting specific epigenetic readers (e.g., BET inhibitors) and writers (e.g., METTL3 inhibitors) are under investigation. For example, STM2457 can precisely target METTL3, minimizing effects on other methyltransferases (103). As the first METTL3 inhibitor to enter clinical evaluation (NCT05584111), STC-15 demonstrates transformative potential in translating epitranscriptomic discoveries into therapeutic applications (104). These inhibitors have demonstrated significant efficacy in suppressing tumor cell proliferation, migration, and invasion, particularly in GC.

In therapeutic development, current epigenetic therapies need optimization through precision delivery systems such as METTL3 siRNA-loaded nanoparticles, rational combination strategies like HDAC inhibitors with immunotherapy, and innovative approaches including CRISPR-dCas9-mediated targeted demethylation to address tumor heterogeneity (105, 106). A new research shows an innovative diagnostic and therapeutic 808 nm NIR laser irradiation-triggered engineered microbe that establishes an efficient delivery platform for DNMT1 inhibitors and iMXene, activating epigenetic immunity and paving the way for the broad application of epigenetic regulation in solid tumor treatment (107).

The translation of epigenetic biomarkers requires rigorous clinical validation. Future research should prioritize the identification and validation of biomarkers with well-defined mechanisms and clear clinical relevance. Successful clinical implementation will depend on multidisciplinary collaboration across molecular biology, materials science and bioinformatics to establish GC-specific epigenetic databases and advance single-cell epigenomic technologies, while developing ethical guidelines for emerging technologies like epigenetic editing. The future of GC epigenetics lies in developing clinically validated high-sensitivity detection systems, implementing multi-omics-guided precision therapy, and building cross-disciplinary innovation ecosystems. As the field transitions from mechanistic discovery to clinical implementation, these advances promise to revolutionize early detection and personalized treatment, ultimately achieving precision medicine for GC patients through sustained collaboration across academia, clinical practice and industry. Advances in ChIP, CUT&Tag, and high-resolution sequencing are profoundly transforming GC epigenetics. Moving forward, integrating multi-omics data across molecular, cellular, and tissue levels will systematically uncover the core functions of epigenetic regulation, translating these insights into clinical predictive models and precision therapies.

Future directions should focus on: (1) elucidating synergistic epigenetic mechanisms to refine diagnostic models; (2) developing real-time liquid biopsy platforms for treatment monitoring and recurrence surveillance; (3) implementing precision medicine approaches through integrated epigenetic profiling. These advances will likely transform GC management from reactive treatment to proactive interception.

## 8 Conclusion

As summarized in Table 1, the past 6 years have yielded a wealth of candidate epigenetic biomarkers for GC. However, it is clear that not all are created equal. From a translational perspective, DNA methylation markers (particularly RNF180/Septin9) currently hold the most immediate promise for early detection, as evidenced by their regulatory approval and inclusion in clinical guidelines. Non-coding RNAs like miR-21 offer robustness due to their high abundance, while key RNA modification regulators (e.g., METTL3) represent compelling targets for both therapy and companion diagnostics (108, 109). Despite this progress, the field’s primary challenge is no longer discovery but validation and integration. Current liquid biopsy approaches based on single markers still lack the sensitivity for reliable early-stage detection. Therefore, we argue that the most constructive path forward is the development of integrated multi-omics liquid biopsy panels that combine the best-in-class epigenetic markers (e.g., RNF180 methylation) with other analyte types (e.g., fragmentomics, proteomics) to achieve the necessary diagnostic performance.

**TABLE 1:** Representative epigenetic biomarkers in gastric cancer.

Biomarker type	Specific marker	GC stage/context	Potential clinical application	Merit/evidence level
DNA methylation	RNF180/septin9	Early GC/high-risk screening	Early detection	★★★★★ (NMPA-approved kit; expert consensus endorsement)
	APC/MINT31	Early GC/precancerous lesions	Early detection/risk stratification	★★★★☆ (Consistently reported in cohorts)
	SPG20/FBN1	Advanced GC	Prognosis (poor survival)	★★★★☆ (Strong correlation in multiple studies)
	CPNE1	Advanced GC (receiving chemo)	Predictive (chemotherapy response)	★★★★☆ (Identified in comprehensive analyses)
	20-CpG signature	Advanced GC (receiving anti-PD-1)	Predictive (immunotherapy response)	★★★☆☆ (Promising initial data [37])
Histone modification	H3K18la	Advanced GC (metabolic subtype)	Prognosis (poor survival, immune evasion)	★★★☆☆ (Mechanistically strong; direct detection challenging)
	HDAC overexpression	Advanced GC	Prognosis/predictive (immunotherapy?)	★★★☆☆ (Correlative data; targeted therapies exist)
Non-coding RNA	miR-21	Various stages	Diagnosis/prognosis	★★★★☆ (Extensively validated; high abundance)
	HOTAIR	Various stages	Prognosis (metastasis)	★★★☆☆ (Strong functional data)
	circPVT1	Various stages	Diagnosis/prognosis	★★★☆☆ (Stable in fluids; stage-specific expression)
RNA modification	METTL3	Advanced GC	Prognosis (poor survival)	★★★★☆ (Consistent overexpression; pro-tumorigenic function)
	METTL14	Advanced GC	Prognosis (better survival)	★★★☆☆ (Consistently reported)
	YTHDF1	Advanced GC	Prognosis (poor survival)	★★★★☆ (Critical reader; drives progression)
	YTHDF2	Advanced GC	Predictive (chemotherapy resistance)	★★★☆☆ (Reported)
	FTO	Advanced GC	Prognosis (metastasis)	★★★☆☆ (Pro-metastatic; potential target)
	METTL1	Advanced GC	Prognosis (poor survival)	★★★☆☆ (Reported)
	NSUN2	Early GC/precancerous lesions	Early detection/risk stratification	★★★☆☆ (Strong functional data)
	NAT10	Early GC/precancerous lesions	Early detection/risk stratification	★★★★☆(Strong functional data)

## Glossary

GC: Gastric Cancer
DNMT: DNA Methyltransferase
5-mC: 5-Methylcytosine
*H. pylori*: *Helicobacter pylori*
EBV: Epstein-Barr Virus
HER2: Human Epidermal Growth Factor Receptor 2
TCGA: The Cancer Genome Atlas
MSI: Microsatellite Instability
PD-L1: Programmed Death-Ligand 1
p16: Multiple tumor suppressor 1
MLH1: MutL homolog 1
APC: Adenomatous Polyposis Coli
Wnt/β-catenin: Wingless-related integration site/Beta-catenin
CHD: Chromodomain Helicase DNA-Binding Protein 1
NF-κB: Nuclear Factor Kappa B
PI3K/AKT: Phosphoinositide 3-Kinase/Protein Kinase B
MAPK1: Mitogen-activated protein kinase
CDK1: Cyclin-dependent kinase 1
RUNX3: Runt-related transcription factor 3
TIMP3: Metalloproteinase inhibitor 3
PTEN: Phosphatase and tensin homolog
ctDNA: Circulating Tumor DNA
RNF180: Ring finger protein 180
NMPA: National Medical Products Administration
SPG20: Spartin
FBN1: Fibrillin-1
CPNE1: Copine-1
EV: Extracellular Vesicle
H3K18la: Histone H3 Lysine 18 Lactylation
VCAM1-AKT: Vascular cell adhesion molecule1-protein kinase B
p21: Cyclin-dependent kinase inhibitor 1A
HDAC: Histone Deacetylase
EZH2: Enhancer of Zeste Homolog 2
KDM6A/B: Lysine Demethylase 6A/B
LSD1: Lysine-Specific Demethylase 1
SETD1A: SET domain containing 1A
HIF1α: Hypoxia inducible factor 1
SUMO: Small ubiquitin-like modifier
ADP: Adenosine diphosphate
ChIP-seq: Chromatin Immunoprecipitation Sequencing
CUT&Tag: Cleavage Under Targets and Tagmentation
CUT&Tag-BS: CUT&Tag with Bisulfite Sequencing
SWI/SNF: Switch/Sucrose Non-Fermentable (chromatin remodeling complex)
CHD: Chromodomain helicase DNA binding protein
ARID1A: AT-Rich Interaction Domain 1A
3′-UTR: 3′untranslated region
EMT: Epithelial-Mesenchymal Transition
ncRNA: Non-Coding RNA
miRNA: MicroRNA
lncRNA: Long Non-Coding RNA
circRNA: Circular RNA
ceRNA: Competing Endogenous RNA
TSPEAR-AS2: TSPEAR antisense RNA 2
CCND1: Cyclin D1
SRPK1: Serine/arginine protein kinase 1
hnRNPA2B1: Heterogeneous nuclear ribonucleoprotein A2/B1
RON: Macrophage stimulating 1 receptor,MST1R
m6A: N6-Methyladenosine
m7G: N7-Methylguanosine
m5C: 5-Methylcytosine (RNA modification)
ac4C: N4-Acetylcytidine
METTL1/3/14/16: Methyltransferase-like 1/3/14/16
YTHDF1/2: YTH N6-Methyladenosine RNA Binding Protein 1/2
STAT5A: Signal transducer and activator of transcription 5A
DDX21: DEAD (Asp-Glu-Ala-Asp) box helicase 21
FDX1: Ferredoxin 1
USP14: Ubiquitin-Specific-Processing Protease 14
ONECUT2: One cut homeobox two
IGF2BP2: Insulin-like growth factor 2 mRNA-binding protein 2
CSF2: Colony stimulating factor 2
MSC: Mesenchymal Stem Cell
FTO: Fat Mass and Obesity-Associated Protein
ALKBH5: AlkB Homolog 5
PKMYT1: Protein kinase, membrane associated tyrosine/threonine 1
WRAP53: WD repeat containing antisense to TP53
JAK1: Janus kinase 1
WDR4: WD repeat domain 4
NSUN2: NOP2/Sun RNA Methyltransferase 2
NAT10: N-Acetyltransferase 10
MDM2: Murine double minute2
HK2: Hexokinase 2
SEPT9: Septin 9
BET: Bromodomain and Extra-Terminal (protein family)
CRISPR/dCas9: Clustered Regularly Interspaced Short Palindromic Repeats/deactivated Cas9
NIR: Near Infrared
